# Morphological adaptations in breast cancer cells as a function of prolonged passaging on compliant substrates

**DOI:** 10.1371/journal.pone.0187853

**Published:** 2017-11-14

**Authors:** Sana Syed, Joseph Schober, Alexandra Blanco, Silviya Petrova Zustiak

**Affiliations:** 1 Department of Biomedical Engineering, Saint Louis University, Saint Louis, Missouri, United States of America; 2 Department of Pharmaceutical Sciences, Southern Illinois University Edwardsville, Edwardsville, Illinois, United States of America; University of South Carolina, UNITED STATES

## Abstract

Standard tissue culture practices involve propagating cells on tissue culture polystyrene (TCP) dishes, which are flat, 2-dimensional (2D) and orders of magnitude stiffer than most tissues in the body. Such simplified conditions lead to phenotypical cell changes and altered cell behaviors. Hence, much research has been focused on developing novel biomaterials and culture conditions that more closely emulate *in vivo* cell microenvironments. In particular, biomaterial stiffness has emerged as a key property that greatly affects cell behaviors such as adhesion, morphology, proliferation and motility among others. Here we ask whether cells that have been conditioned to TCP, would still show significant dependence on substrate stiffness if they are first pre-adapted to a more physiologically relevant environment. We used two commonly utilized breast cancer cell lines, namely MDA-MB-231 and MCF-7, and examined the effect of prolonged cell culturing on polyacrylamide substrates of varying compliance. We followed changes in cell adhesion, proliferation, shape factor, spreading area and spreading rate. After pre-adaptation, we noted diminished differences in cell behaviors when comparing between soft (1 kPa) and stiff (103 kPa) gels as well as rigid TCP control. Prolonged culturing of cells on complaint substrates further influenced responses of pre-adapted cells when transferred back to TCP. Our results have implications for the study of stiffness-dependent cell behaviors and indicate that cell pre-adaptation to the substrate needs consideration.

## Introduction

Standard tissue culture practices, which involve propagating cells in serum-containing cell culture medium on tissue culture polystyrene (TCP) dishes, which are flat, 2-dimensional (2D) and orders of magnitude stiffer than most tissues in the body [1], have been employed for over a decade [2]. While extremely useful due to their affordability, convenience, biocompatibility, and robustness [3–5], mounting research evidence suggests that the simplified conditions could also lead to very different cell responses compared to the *in vivo* cell environment [6]. Biomaterials capable of emulating one or several aspects of cell microenvironments are, thus, gaining importance as bridges between standard tissue culture and *in vivo* conditions [7]. Consequently, a question that has emerged is how long does it take for cells, and in particular cell lines propagated on TCP surfaces, to adapt to their new biomaterial environment and what would the implications of such an adaptation be.

To begin answering this question, here we focus on substrate stiffness, which is one of several major biomaterial properties that significantly affect cell behaviors. Importantly, substrate stiffness can be controlled readily and reproducibly [8–10] and cell responses to substrate stiffness have been well-documented [11, 12]. For example, substrate stiffness has been shown to affect cell viability, growth, and proliferation [13], cell morphology, cytoskeletal structure and adhesion [14], stem cell differentiation [15], cell migration [16, 17] and drug responsiveness [18, 19] among other properties. Interestingly, for much of the work related to stiffness-dependent cell responses, cells have been continuously passaged on TCP, then seeded onto selected compliant substrates and tested for cell responses without pre-adapting the cells to their new microenvironment beyond several hours [20, 21].

In this work, we hypothesized that: *i)* cell behaviors would change as a function of a prolonged pre-adaptation to a compliant substrate, and *ii)* upon adaptation, the differences in certain cell behaviors on soft *versus* stiff substrates will be minimized. Our hypothesis is based on the premise that most cells, and in particular cell lines, have adapted to stiff environment during continuous passaging on TCP, regardless of the stiffness of their tissue of origin. The effect of continuous cell passaging on stiff TCP on cell properties has been widely studied over the past decade [22]. For example, after primary porcine coronary arterial endothelial cells were passaged *in vitro* on TCP for multiple passages, they exhibited reduced proliferative capacity, increased oxidation, enhanced apoptosis, and even activation of p53 signaling pathways, which is a pathway that plays a significant role in tumor metastasis [22, 23]. Another study examined long-term effects of *in vitro* passaging of primate brain micro-vessel endothelial cells, showing that it caused changes in enzyme activities and in the total protein content [24]. Further, a study conducted on human umbilical vein endothelial cells showed alterations in expression of actin and cytoskeletal proteins, as well as changes in cell spreading, migration, and shape after serial passaging of cells *in vitro* on TCP [25]. Although studies have looked into the effects of prolonged passaging on TCP, the effect of prolonged cell passaging on biomaterials, specifically for cell lines that have been conditioned to TCP, has generally been overlooked.

To address this gap, we cultured two breast cancer cell lines on polyacrylamide (PA) hydrogels of varying stiffness (1–103 kPa; TCP was used as control) for 9 consecutive days or 3 passages. We then studied cell morphology, adhesion, proliferation and spreading rate as a function of cell adaptation to the stiffness of the underlying substrate. We observed significant adaptation upon prolonged passaging, which resulted in diminished differences in cell behaviors on soft *versus* stiff substrates. Cells exhibited increased adhesion, proliferation and spreading at day 9 as compared to day 1 of a prolonged culture on soft PA gels. Our results have considerable implications for future studies on stiffness-dependent cell responses and underscore the need for cell pre-adaptation to the substrate.

## Materials and methods

All materials were acquired from Fisher Scientific (Waltham, MA), unless noted otherwise.

### Preparation and collagen coating of polyacrylamide hydrogels

Gel precursor solutions were prepared by mixing acrylamide (40% w/v, Bio-Rad, Hercules, CA) and the crosslinker bisacrylamide (bis; 2% w/v, Bio-Rad, Hercules, CA) and de-ionized water (DI water) in a 15 mL conical for a total volume of 5 ml (Table 1). Precursor solutions were mixed well and placed in a degassing chamber (BelArt, Wayne, NJ) for 30 min. Then 25 μL of 10% w/v ammonium persulfate (APS; Bio-Rad, Hercules, CA) and 2.5 μL of tetramethylethylenediamine (TEMED, Sigma-Aldrich, St. Louis, MO) were added, and the gel precursor solution was gently mixed by pipetting up and down. The solution was then pipetted onto a glass slide pre-treated with Repel Silane^®^ (GE Healthcare Life Sciences, Logan, UT). Silicone spacers (1 mm thickness; GE Healthcare, Little Chalfont, UK) were placed at the edges of the glass slide and a flexible plastic support (GelBond PAG Film, GE Healthcare Life Sciences, Pittsburg, PA) was placed on top, hydrophilic side-down. GelBond enables polyacrylamide (PA) hydrogels to adhere to it permanently upon polymerization for easy gel handling. The hydrogel precursor assembly was then placed in a degassing chamber for 45 min to polymerize. Once formed, gels were secured to the bottom of a tissue culture dish (multiwell plates or petri dishes) using polydimethylsiloxane (PDMS; Sylgard 182 Elastomer Kit, Elsworth Adhesives, Germantown, WI) and then placed at 37°C for 2 h to allow the PDMS to cure. The PA gels were then soaked in DI water at room temperature for 24 h to allow swelling and release of unreacted toxic monomers.

**Table 1:** Composition and Young’s moduli of polyacrylamide gels.

Acrylamide %	Bis-acrylamide %	Acrylamide from 40% stock solution (mL)	Bis-acrylamide from 2% stock solution (mL)	DI Water (mL)	E ± St. Dev. (kPa)
3	0.060	0.375	0.150	4.475	0.48 ± 0.16
5	0.025	0.625	0.063	4.312	1.06 ± 0.27
5	0.150	0.625	0.375	4.000	4.47 ± 0.58
8	0.250	1.000	0.625	3.375	32.60 ± 0.10
12	0.250	1.500	0.625	2.875	102.52 ± 28.79

The PA gels were coated with 0.2 mg/mL Rat Tail Collagen Type I (BD Biosciences, San Jose, CA) via sulfosuccinimidyl-6-(4'-azido-2'-nitrophenylamino hexanoate) (sulfo-SANPAH; Sigma-Aldrich St. Louis, MO) crosslinker (4% w/v in a solution of 4 part dimethyl sulfoxide (DMSO) and 96 parts DI water). Briefly, sulfo-SANPAH was pipetted on PA gels to cover the surface, and the gels were placed inside an ultraviolet (UV) oven (UV1080, UVITRON, West Springfield, MA) for 300 sec (intensity of 67 mW/cm^2^). Gels were then rinsed with DI water 2 times. Collagen solution was added on top of each gel and allowed to react for 2 h at room temperature. The excess unreacted collagen was rinsed with 1X phosphate buffered saline (PBS) and gels were sterilized under UV (~302 nm) in a tissue culture hood for 2 h. Collagen-coated PA gels were soaked in 1X RPMI medium (GE Healthcare Hyclone, Little Chalfont, UK) supplemented with 10% fetal bovine serum (FBS; GE Healthcare Hyclone, Little Chalfont, UK) and 1% penicillin/streptomycin (P/S; MP Biomedicals, Santa Ann, CA) for at least 4 h immediately prior to use.

### Rheological testing

Hydrogel samples were prepared by depositing 500 μL of PA gel precursor solution between two Repel-Silane-treated glass plates spaced 2 mm apart with silicone spacers to create even thickness gels. After polymerization, gels were allowed to swell for 24 h in DI water and then cut into a circle with a diameter of 20 mm using a cookie cutter. Gel stiffness was measured by rheology (AR 2000ex rheometer, TA Instruments, New Castle, Delaware) with a 20 mm upper parallel plate geometry, oscillatory frequency sweep test of 1–10 Hz, and a 2% constant strain. Young’s modulus was related to the storage modulus by the following equation:

$$E=G^{\prime }2(1+v)$$ (1)

where *E* is Young’s modulus and *v* is the Poisson’s ratio (0.5 for PA gels [26]).

### Cell culture and maintenance

MDA-MB-231 and MCF-7 human breast cancer cells (obtained from NCI-DCTD Repository, NCI, Frederick, MD) were cultured in RPMI medium supplemented with 10% FBS and 1% P/S in a humidified incubator at 37°C and 5% CO_2_. Cells were passaged after reaching ~80% confluency (~every 5–6 d) and media was changed every 2 d. Only cells up to passage 15 were used for experiments. Cells were harvested by a 5 min exposure to 0.5% trypsin (Sigma-Aldrich, St. Louis, MO) and seeded onto PA gels. Cells were cultured continuously for 9 d on PA gels and TCP control, or re-passaged every 3 d for a total of 9 d (i.e. 3 passages). For the continuous 9 d culture, initial cell seeding density was 5x10^4^ cells/ml, while for the latter experiment, where cells were re-passaged every 3 d, the initial cell seeing density was 1x10^5^ cells/ml (for every passage). Media was changed every 2 d for all conditions.

### Cell attachment and proliferation analysis

Cell attachment efficiency on PA gels and TCP control was tested by counting cells after 4 h and 24 h of passage 1 (P1), and then after 24 h of passage 2 (P2), and again after 24 h of passage 3 (P3). To do so, first, media was removed and the cells on PA gels or TCP were gently rinsed with 1X PBS to remove loosely attached cells. The attached cells were harvested via Trypsin and counted with a Brightline^™^ Phase hemocytometer (Fisher Scientific, Waltham, MA) under an inverted microscope. Cell attachment efficiency was calculated as (note that cell proliferation was considered negligible at tested times “*t*”):

$$Attachment\;Efficiency\;(\%)=(\frac{Cell\;Number\;at\;time\;t}{Cell\;number\;at\;seeding})100.$$ (2)

Cell proliferation (i.e. fold-change in cell number) was expressed as:

$$Cell\;Proliferation=\frac{Cell\;number\;at\;72\;h}{Cell\;number\;at\;24\;h}.$$ (3)

### Cell morphology analysis

To determine cell morphology (cell spreading area and cell circularity) as a function of prolonged culture on PA gels of 1 and 103 kPa (TCP control also used in all experiments), cells were imaged every day for a total of 9 d. Again, there were two experimental conditions, where cells were either re-passaged every 3 d for a total of 3 passages, or cultured on the same substrate for the duration of the 9 d culture. Brightfield images were taken using a light microscope (Axiovert 200 M, Zeiss, Oberkochen, Germany) with Axiovision software at a magnification of 20X and analyzed on ImageJ (NIH public domain software) using Shape Descriptor macros. Shape factor (a measure of circularity) was defined as:

$$Shape\;Factor=\frac{4\pi (area)}{{Perimeter}^{2}}.$$ (4)

This formula gives a number from 0–1, where values of 0.6–1 indicated rounded cells and values of 0–0.5 indicated elongated cells. A perfect circle would have a value of 1 and a perfect line a value of 0.

### Live imaging of cells

Live cell imaging was performed using Leica Microsystem DMi8 (Leica, Wetzler, Germany) and the MetaMorph acquisition and image analysis software (Molecular Devices, Sunnyvale, CA). PA gels were prepared as described above. Collagen-coated 18 mm glass coverslips were used as control. PA gels or glass coverslips were placed at the center of a glass bottom, 0.17 mm Delta T dish (Bioptechs Inc, Butler, PA) [27]. Cells were then seeded on top of the PA gels (or glass coverslip control) at 1x10^5^ cells/ml and submerged in RPMI medium supplemented with 10% FBS and 25 mM HEPES sodium salt (Sigma Aldrich, Saint Louis, MO) of pH 7.4. Subsequently, the Delta T dish was placed onto the microscope’s Delta T 4 stage adapter for DMIR Leica Microscope (Bioptechs, Butler, PA) to begin live cell imaging. A 20X objective was used. Imaging was set up to take brightfield images every 15 sec for 5 h. Images were compiled to create an .AVI file on ImageJ and used individually to analyze spreading area as well as cell spreading rate calculated as:

$$\text{Spreading}\;\text{Rate}=(\frac{{Cell\;Area}_{final}-{Cell\;Area}_{initial}}{{Time}_{final}-{Time}_{initial}}).$$ (5)

The following conditions were tested: cells collected from 1 kPa P3 and seeded onto a new 1 kPa gel (1→1); cells collected from 103 kPa P3 and seeded onto a new 103 kPa gel (103→103); TCP control, i.e. cells collected from TCP and seeded on a collagen-coated glass coverslip (TCP→TCP); cells collected from TCP and seeded onto a 1 kPa gel (TCP→1); and cells collected from TCP and seeded onto a 103 kPa gel (TCP→103).

### Statistical analysis

A minimum of 3–6 independent experiments were conducted for each condition. For all cell analysis, a minimum of 900 cells were analyzed (minimum of 3 independent experiments, 3 samples per experiment, 3 images per sample; ~100 cells per sample). Results are reported as averages ± standard deviation. Student t-test was performed to compare between two samples and ANOVA with a Tukey’s post-hoc test was performed to compare between multiple samples, where p<0.05 was considered significant.

## Results

### Stiffness-dependent morphology of MDA-MB-231 cells

Five PA gels of varying stiffness (0.5–102.5 kPa) were prepared as described in Table 1 and detailed in the Methods section. Storage modulus was measured via rheology and converted to Young’s modulus as per Eq 1; Young’s modulus for each gel is reported in Table 1. The stiffness range of ~0.5–102.5 kPa was chosen to encompass the stiffness of all soft tissues in the body [1].

**Fig 1:**
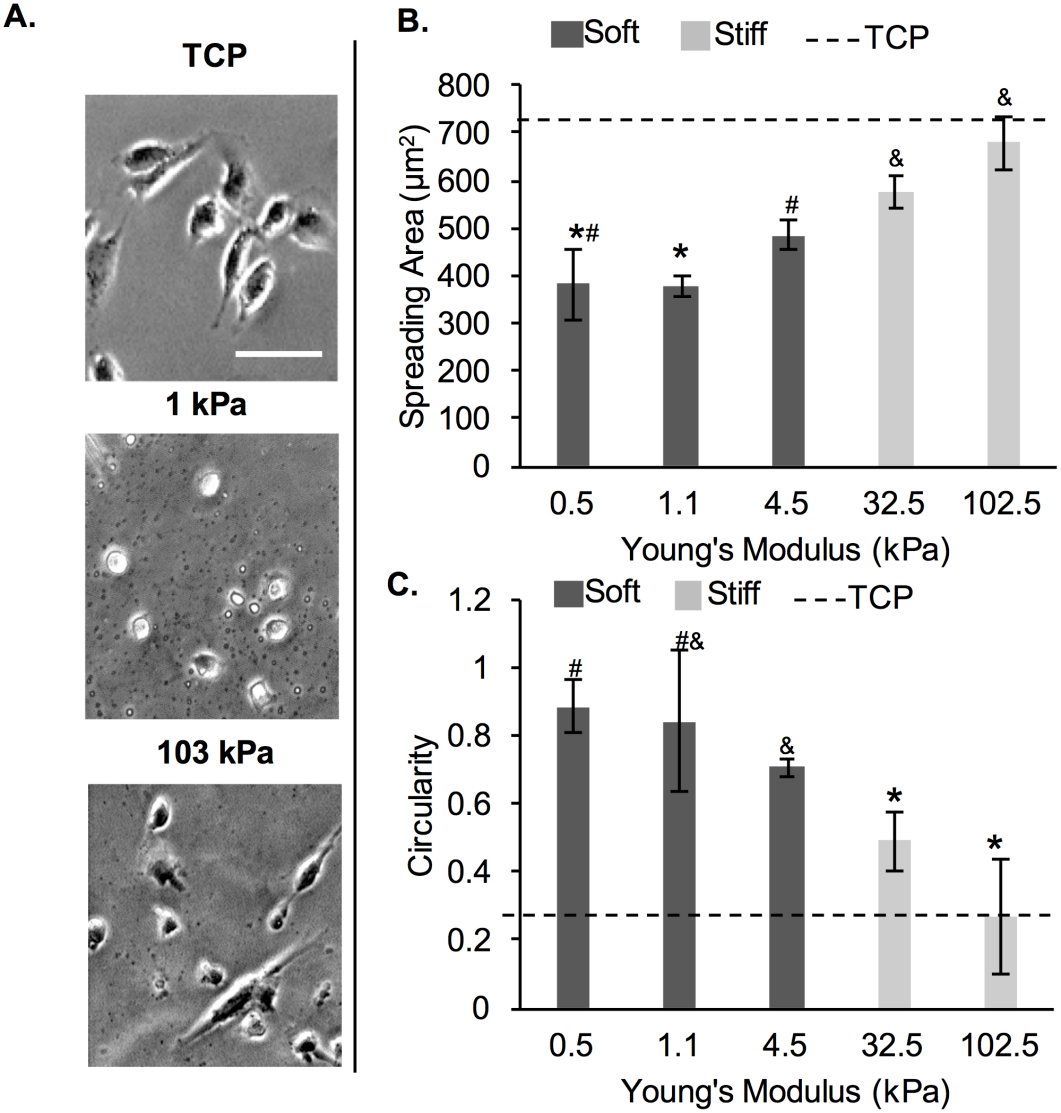
MDA-MB-231 cells on PA gels of varying stiffness. (A) Representative phase contrast cell images taken at 24 h post-seeding. Scale bar is 50 μm. (B) Cell spreading area and (C) circularity at 24 h post-seeding. The dashed line represents values on TCP. *^,#,$^ represent significant difference from each other, where same symbol represents no difference (p<0.05, n = 3).

Here, we determined MDA-MB-231 breast cancer cell morphology in terms of cell spreading area and cell circularity as a function of substrate stiffness at 24 h post-seeding. Representative cell images are shown on Fig 1A. Overall, cells exhibited lower spreading and higher circularity on the softer ~0.5–4.5 kPa gels (dark gray, Fig 1B and 1C), than the stiffer 32.5–102.5 kPa gels (light gray, Fig 1B and 1C). There was no significant difference in spreading area or cell circularity of MDA-MB-231 cells among the 3 soft gels, or between the 2 stiff gels. However, cell spreading area on the 0.5 and 1.1 kPa gels was significantly different from cell area on the 32.5 kPa gels (by ~34%) and from cell area on the 102.5 kPa gels (by ~44%). Cell area on the 4.5 kPa gels was significantly different only from the cell area on the 102.5 kPa gels by ~28%. Cell circularity was significantly different between the cells on 0.5 and 1.1 kPa gels and cells on 32.5 kPa gels (by ~40%) and cells on 102.5 kPa gels (by ~66%). Cell circularity was also significantly different between cells on 4.5 kPa gels and cell on 32.5 kPa gels (by ~30%) and cells on 102.5 kPa gels (by ~60%). For all subsequent experiments, we chose to work with the 1.1 (~1) and 102.5 (~103) kPa gels, since cells showed the most pronounced differences in cell spreading area and circularity between the two conditions. The 1 kPa gels were chosen over the 0.5 kPa gels since cells did not show significant differences in cell morphology between the two conditions and the 1 kPa gels were easier to handle than the 0.5 kPa gels due to improved mechanical stability.

### MDA-MB-231 cell attachment efficiency and cell proliferation as a function of prolonged cell passaging on PA gels

**Fig 2:**
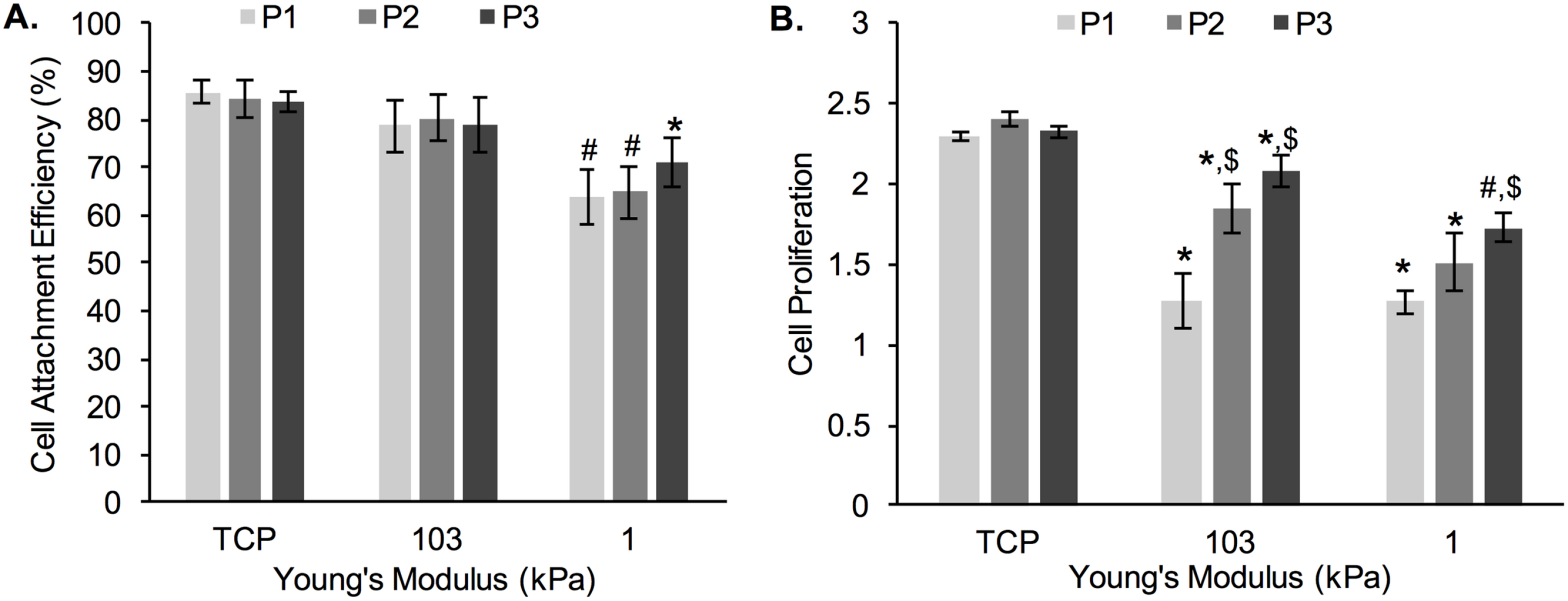
Changes in: A) cell attachment efficiency and B) cell proliferation of MDA-MB-231 cells during prolonged culturing. P1, P2, and P3 designate cell passage numbers. *represent significant difference from TCP, ^#^ represent significant difference from TCP and 103 kPa, ^$^represent significant difference from P1 within the same substrate stiffness (p<0.05, n = 3).

Here, we describe cell attachment efficiency (Fig 2A) and cell proliferation (Fig 2B) as a function of cell re-passaging (i.e. conditioning by continuous passaging) on PA gels of 1 kPa (soft) and 103 kPa (stiff) Young’s modulus. TCP (~3 GPa) was used as control. Our results indicate that cell attachment efficiency for the same substrate remained similar upon passaging (P1 through P3), irrespective of substrate stiffness. However, there was a significant difference between cell attachment efficiency on the 1 kPa gels (~64–70%) and cell attachment efficiency on TCP (~85% for all passages). The average cell attachment efficiency was at ~80% for the 103 kPa gels (for all passages) and was not significantly different than on TCP. Cell attachment efficiency on 1 kPa gels for P1 and P2 was significantly different than cell attachment efficiency on the 103 kPa gels and TCP.

Cell proliferation, calculated as the fold-change in cell number between 24 h and 72 h of culture for each passage (Eq 3), is shown on Fig 2B. Overall, cell proliferation was highest on TCP (at ~2.4-fold change in cell number over 48 h) and remained unaffected by cell re-passaging. In contrast, cell proliferation was significantly lower on both PA gels as compared to TCP, but not significantly different from each other, with the exception of P3, where cell proliferation was ~15% lower on the 1 kPa gels than the 103 kPa gels. Furthermore, cells seeded on the 1 and 103 kPa PA gels showed increase in cell proliferation as a function of cell re-passaging on the same substrate; the increase was more pronounced on the 103 kPa gels, where fold-change in cell number jumped from 1.3-fold to 2.1-fold between P1 and P3, respectively as compared to the 1 kPa gels, where fold-change in cell number jumped from 1.2-fold to 1.7-fold between P1 and P3, respectively.

**Fig 3:**
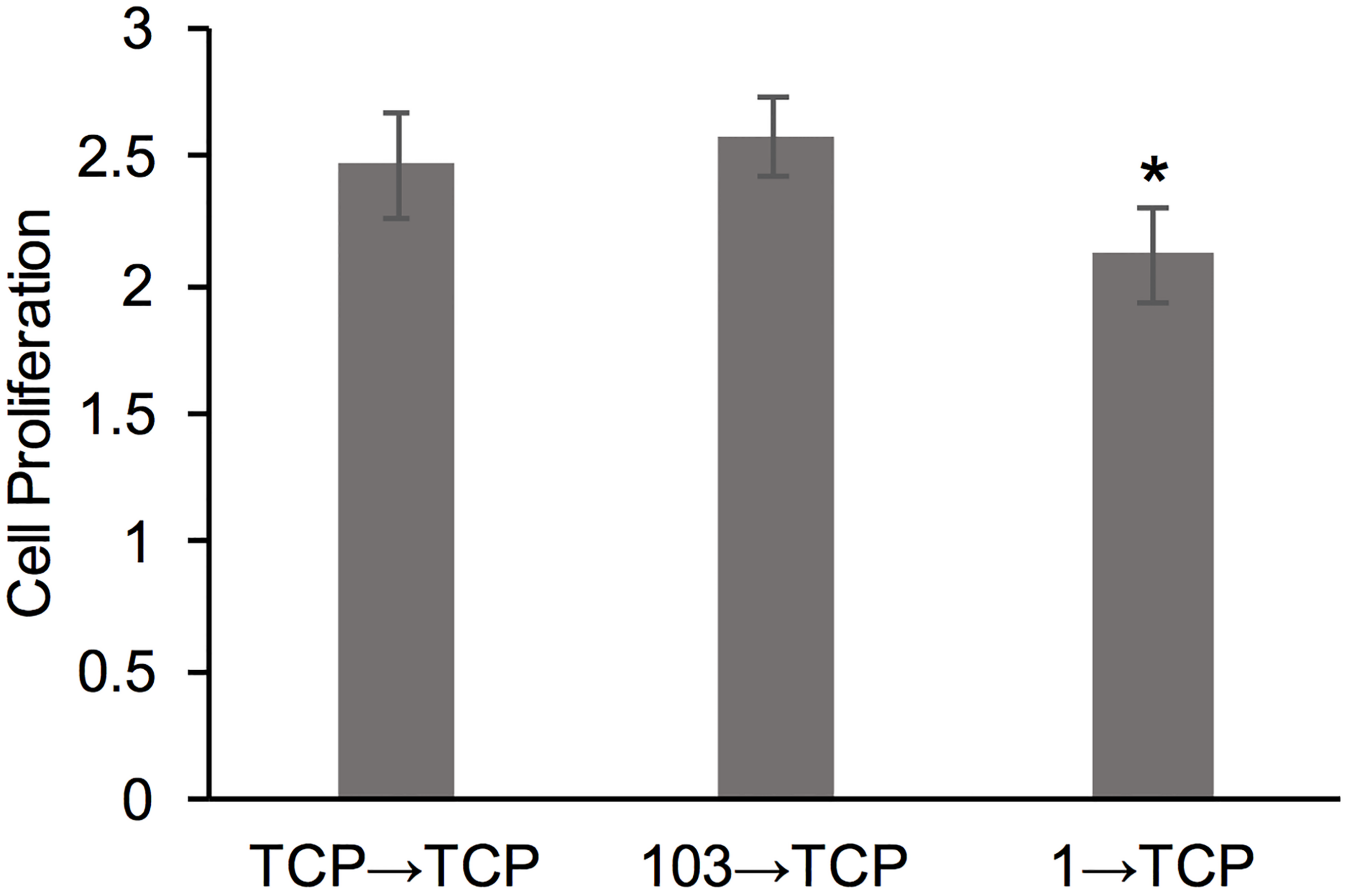
Cell proliferation of MDA-MB-231 cells. Cell proliferation was measured after cells were transferred onto TCP from 3 difference conditions: TCP control (TCP→TCP), P3 on 103 kPa gel (103→TCP), and P3 on 1 kPa gel (1→TCP). *represent significant difference from 103→TCP (p<0.05, n = 3).

To further understand the role of cell adaptation to the substrate on cell proliferation, we harvested cells that have been passaged on TCP, 1 kPa and 103 kPa gels for 3 passages (total of 9 d) and seeded them onto TCP (Fig 3). We noted a significant difference between the 103→TCP and 1→TCP conditions, where the cells re-passaged from the softer gels showed the lowest proliferation. However, all conditions showed a robust proliferation of >2-fold increase in cell number over 48 h.

### MDA-MB-231 cells exhibit morphological changes upon prolonged passaging on PA gels

**Fig 4:**
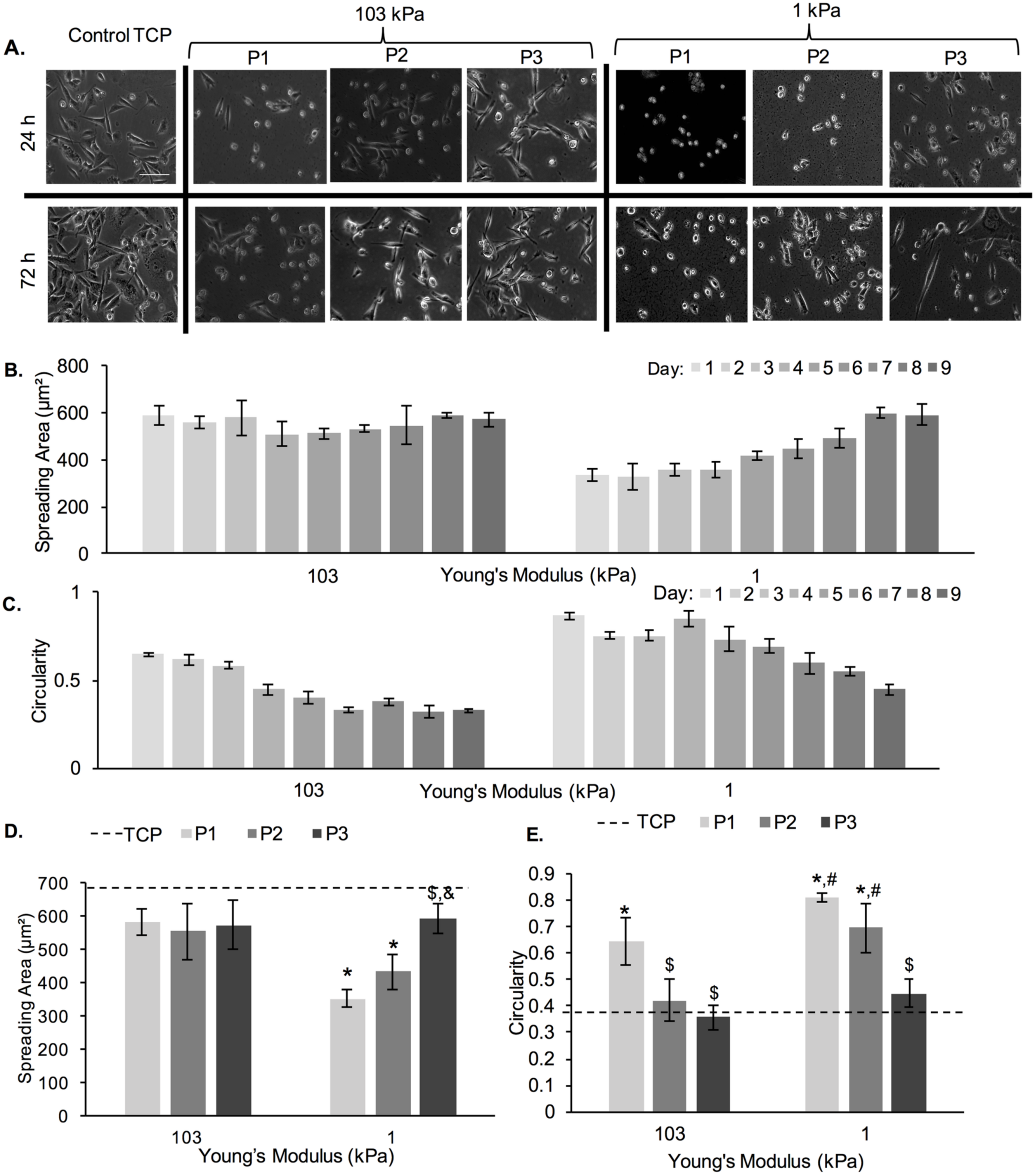
Cell spreading area and circularity of MDA-MB-231 cells as a function of adaptation to substrate stiffness. (A) Representative phase contrast images of MDA-MB-231 cells on 1 kPa and 103 kPa PA gels. Scale bar is 100 μm. (B) Cell spreading area and (C) circularity of MDA-MB-231 cells on 1 kPa and 103 kPa PA gels as a function of culture time. Note that cells were re-passaged every 3^rd^ d for a total of 9 d. (D) Cell spreading area and (E) circularity as measured at 72 h of each passage (P1, P2 and P3). The dashed line represents cell spreading area and circularity on TCP. *represent significant difference from TCP, ^#^represent significant difference from 1 kPa, ^$^represent significant difference from P1 (p<0.05, n = 3).

Fig 4A shows phase contrast images of MDA-MB-231 cells at 72 h of culture for 3 consecutive passages (P1, P2 and P3) on 1 kPa and 103 kPa PA gels (TCP control is also shown). Qualitatively, we noted two trends. First, on the softer 1 kPa gels, we noted more spread cells at 72 h as opposed to 24 h, especially for P2. Second, we noted more spread cells with increase in passage number for cells on both 1 and 103 kPa gels. For all conditions, we noted a number of rounded cells on the hydrogels and minimal number of rounded cells on the TCP control, especially at 24 h of culture. Among all hydrogel conditions, the most number of spread cells was noted on the 103 kPa gels at P3. To quantify these trends, we measured cell spreading area and circularity as a function of time in passage (Fig 4B–4E).

Fig 4B and 4C show spreading area and circularity, respectively, over the course of 9 days, which were subdivided into 3 passages, as previously stated. The cell spreading area on the 103 kPa gels remained relatively constant with no significant changes, while cell circularity decreased by ~0.05/d (from day 1 to day 6). No increase in cell circularity as a function of time was noted for passage 3. Cell spreading area on the 1 kPa gels increased by ~18 μm^2^/d (from day 2 to day 8) and cell circularity decreased by ~0.07/d (from day 4 to day 9). Cell morphology, when compared at 72 h of each passage (Fig 4D and 4E), showed significant differences in circularity and spreading area on 1 kPa PA gels and significant differences in circularity for cells on the 103 kPa gels. Two important trends emerged. First, cell spreading area increased with each passage (for cells on 1 kPa gels) and cell circularity decreased with each passage (for cells on 1 and 103 kPa gels). Second, cell spreading area was larger and cell circularity smaller on 103 kPa gels than on 1 kPa gels for cells of P1, but these differences disappeared at P3. At the end of P3 (day 9), cells on the soft 1 kPa gels looked very similar morphologically to cells on the stiff 103 kPa gels.

**Fig 5:**
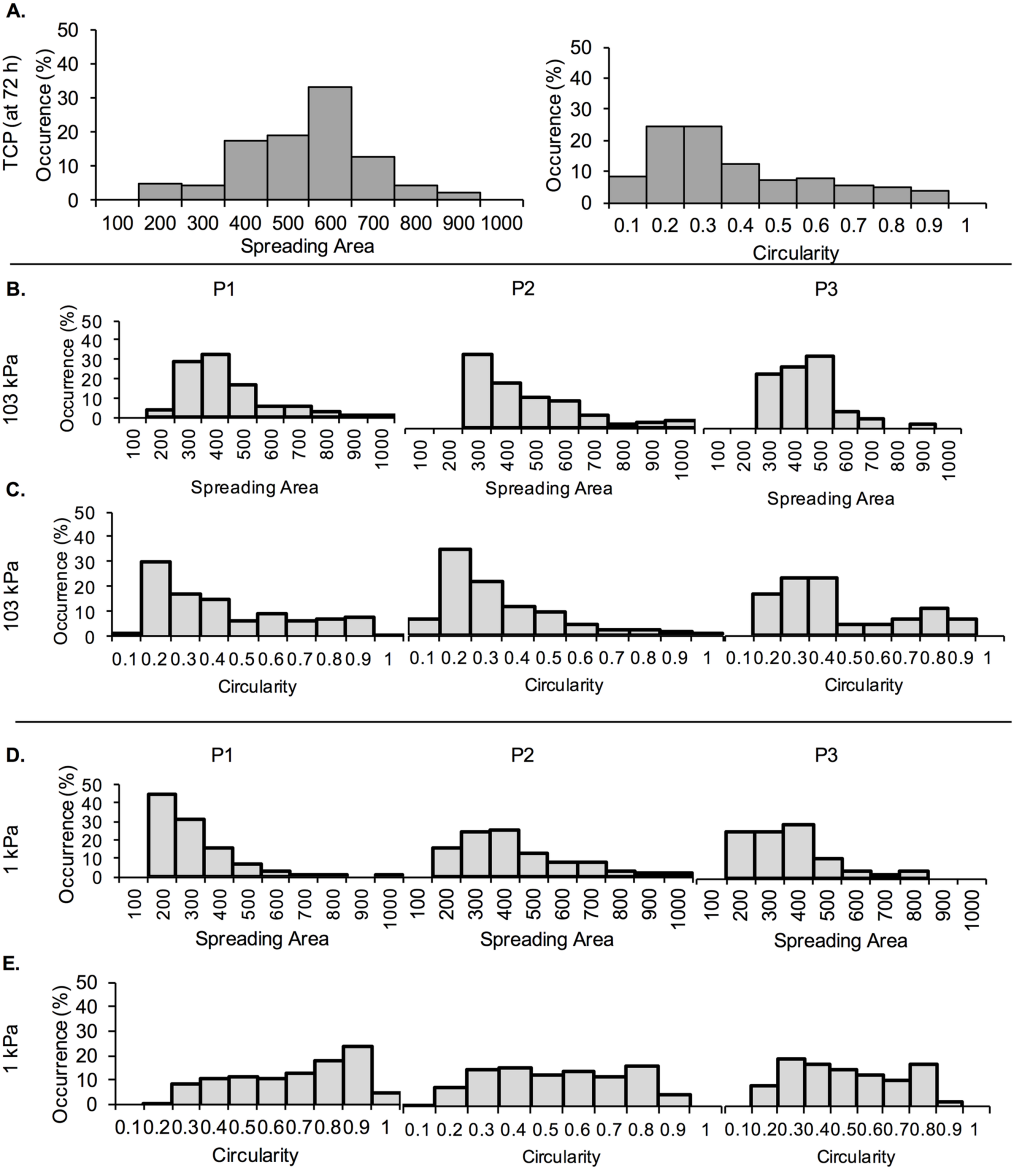
Histograms of MDA-MB-231 cell spreading area and circularity expressed as percent occurrence. Spreading area and circularity were measured at 72 h of each passage (P1, P2 and P3). (A) Percent occurrence of cell spreading area and circularity on TCP. (B) Percent occurrence of cell spreading area and (C) circularity on 103 kPa gels. (D) Percent occurrence of cell spreading area and (E) circularity on 1 kPa gels.

To further characterize the morphology of cells during the three separate passages, percent occurrence of spreading area and circularity values at 72 h were calculated and displayed in histograms (Fig 5). For the TCP control, we noted that most cells had a spreading area of ~600 μm^2^ and circularity of 0.2–0.3 (Fig 5A). The spreading area of cells on the 103 kPa gels shifted very slightly from more cells in the range of 300–400 μm^2^ during P1 and P2, to more cells in the range of 400–500 μm^2^ at P3 (Fig 5B). Circularity also shifted from more cells in the range of 0.2–0.3 during P1 and P2, to more cells in the range of 0.3–0.4 at P3 (Fig 5C). We further noted a large number of cells with smaller (200 μm^2^) spreading area on 1 kPa PA gels for P1, which shifted to 200–400 μm^2^ (400 μm^2^ being the pick) for the later passages (Fig 5D). Correspondingly, the number of cells on 1 kPa PA gels with circularity >0.7 decreased as the passage number increased (Fig 5E).

**Fig 6:**
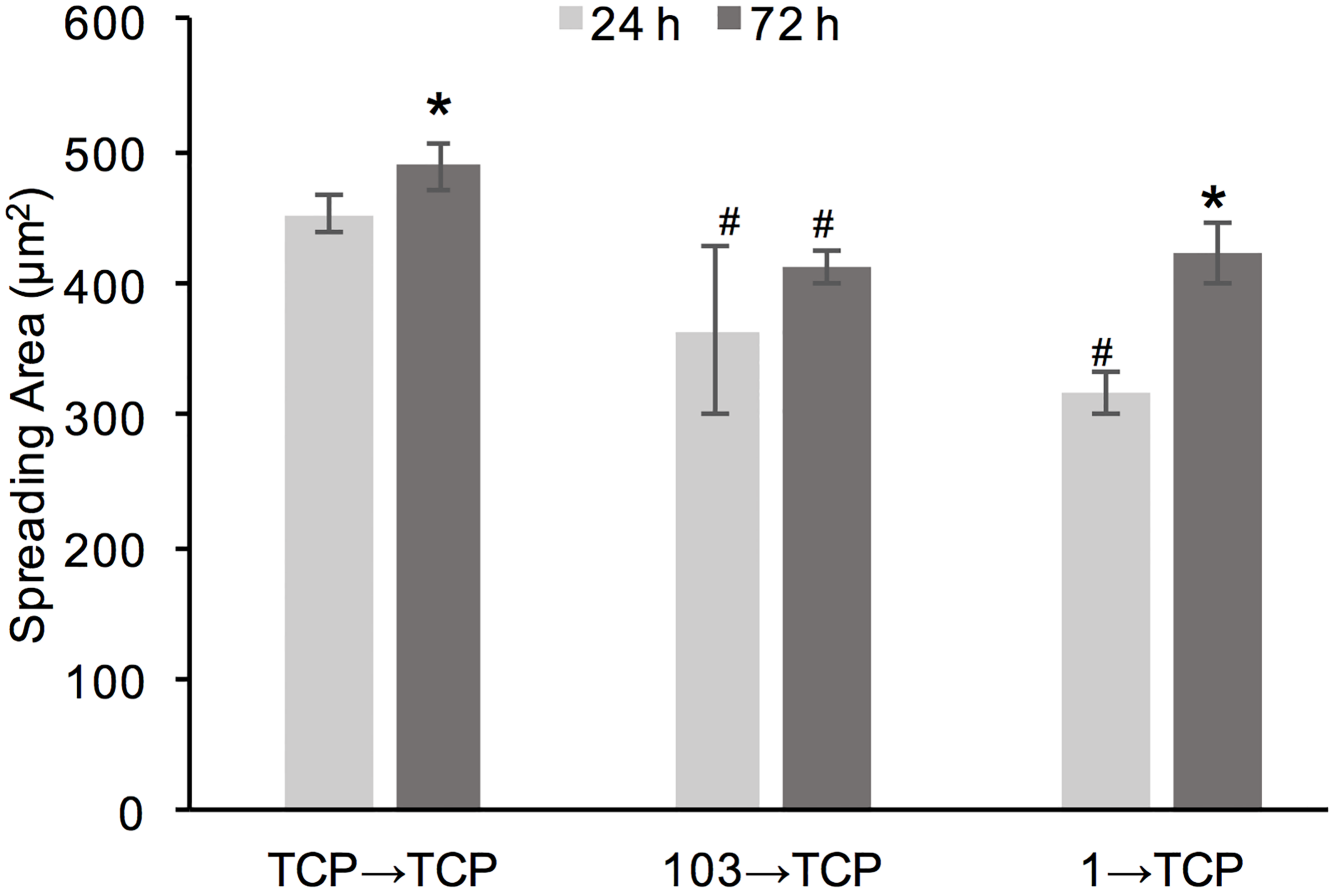
Cell spreading area of MDA-MB-231 cells. Cell spreading area was measured after cells were transferred onto TCP from 3 difference’ conditions: TCP control (TCP→TCP), P3 on 103 kPa gel (103→TCP), and P3 on 1 kPa gel (1→TCP). *represent significant difference from 24 h, ^#^represents significant difference from TCP→TCP (p<0.05, n = 3).

Lastly, MDA-MB-231 cells pre-adapted for 9 d (3 passages) on TCP control, 1 and 103 kPa gels, were passaged on TCP and evaluated for spreading area at 24 h and 72 h (Fig 6). Overall, we saw an increase in spreading area from 24 h to 72 h, which was most significant for the condition 1 →TCP (~26% increase in cell area). At 72 h, both 1→TCP and 103→TCP conditions showed slightly, but significantly, smaller spreading area than the TCP→TCP condition (~14% difference for both); there was no significant difference in cell spreading area between the 1→TCP and 103→TCP conditions.

**Fig 7:**
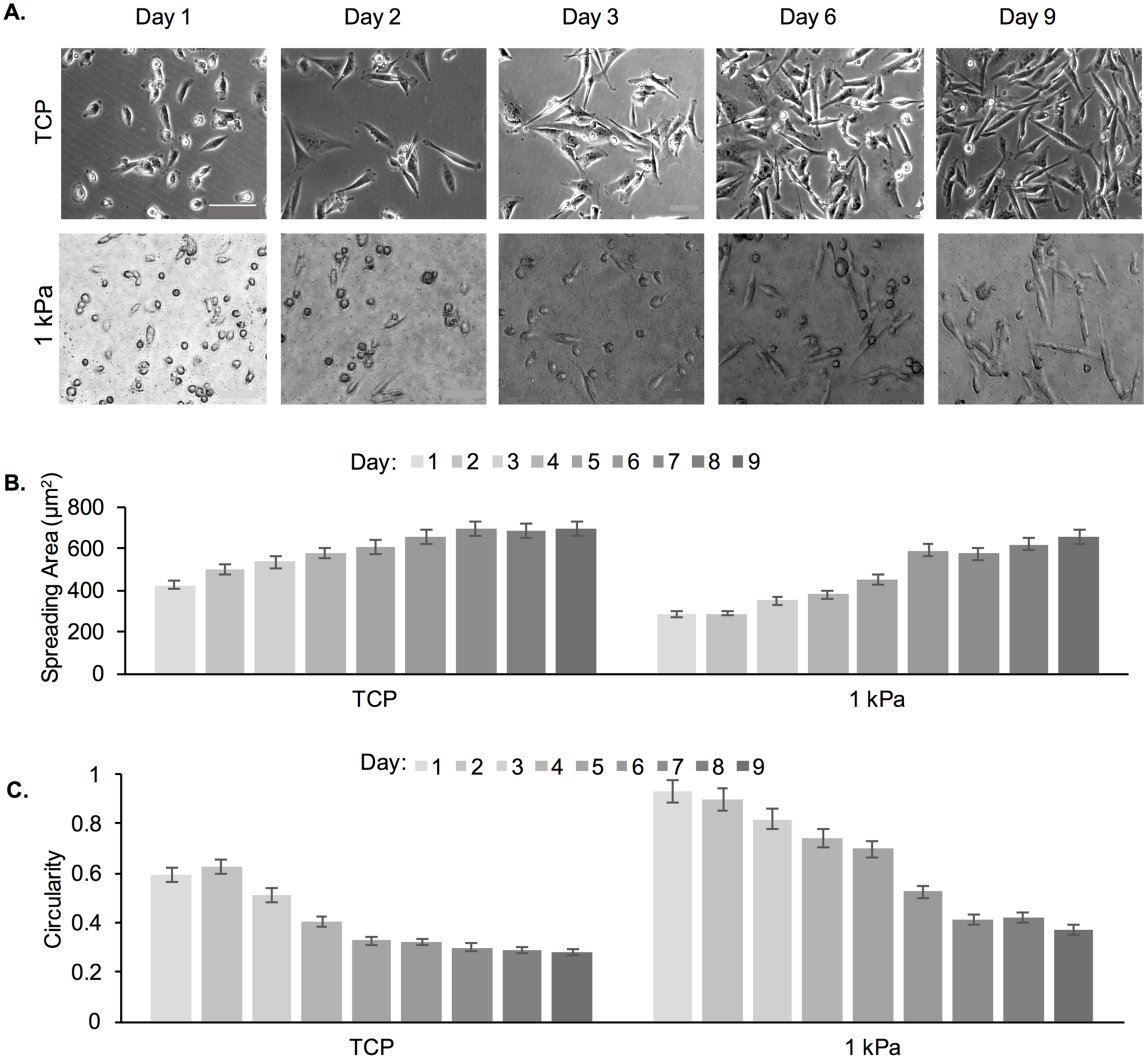
Cell spreading area and circularity of MDA-MB-231 cells as a function of adaptation to substrate stiffness, where cells were seeded onto the PA substrates for 9 consecutive days without re-passaging. (A) Representative phase contrast images of MDA-MB-231 cells on TCP and 1 kPa PA gels. Scale bar is 100 μm. (B) Cell spreading area and (C) circularity of MDA-MB-231 cells on TCP and 1 kPa PA gels as a function of culture time.

We further tested whether similar morphological changes would be observed for cells cultured on a soft substrate (1 kPa gel) for 9 d without re-passaging (Fig 7). TCP was used as a control. The rationale for the continuous 9 d culture was to avoid stress imposed on the cells that results from re-passaging; note that cells were seeded at a lower initial density for this experiment and did not reach >80% confluency at the end of the 9 d culture. Fig 7A shows increased spreading of MDA-MB-231 cells as early as day 3, with more significant numbers of elongated cells at day 6 and day 9. In contrast, while elongated cells were observed on TCP even at day 1, by day 2 most of the cells were elongated. Fig 7B and 7C show increased spreading area and decreased circularity as a function of time for cells on both substrates. When comparing the spreading area of cells in the continuous 9 d experiment *versus* cells that were re-passaged 3 times (for a total of 9 d), we observed that a spreading area of <600 μm^2^ was reached at day 6 for the 9 day continuous experiment compared to day 8 in the multiple passaging experiment.

### MDA-MB-231 cells exhibit changes in cell spreading rate upon prolonged passaging on PA gels

**Fig 8:**
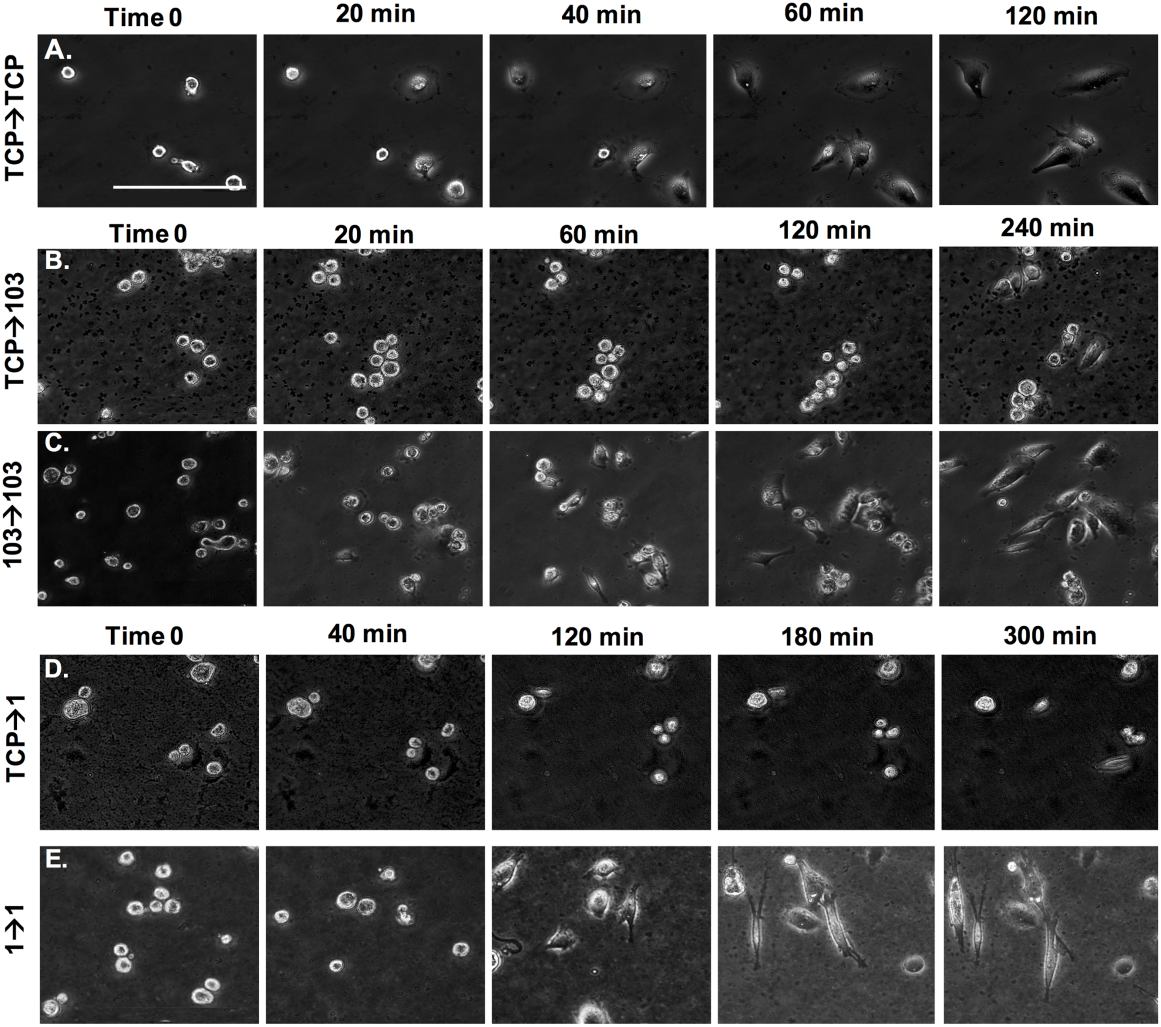
Live imaging of MDA-MB-231 cells on TCP, 1 kPa and 103 kPa PA gels immediately post-seeding on the substrate. (A) Cells were harvested from TCP and seeded onto TCP. (B) Cells were harvested from TCP and seeded onto a 103 kPa gel. (C) Pre-adapted cells at P3 were harvested from a 103 kPa gel and seeded onto a 103 kPa gel. (D) Cells were harvested from TCP and seeded onto a 1 kPa gel. (E) Pre-adapted cells at P3 were harvested from a 1 kPa gel and seeded onto a 1 kPa gel. Scale bar is 100 μm.

To determine if cell spreading rate was impacted by prolonged passaging on PA hydrogel substrates, live imaging of MDA-MB-231 cells was conducted for the following conditions: TCP→1 (cells harvested from TCP and seeded onto a 1 kPa gel), TCP→103 (cells harvested from TCP and seeded onto a 103 kPa gel), TCP→TCP (cells harvested from TCP and seeded onto TCP), 1→1 (cells at P3 harvested from a 1 kPa gel and seeded onto a 1 kPa gel), and 103→103 (cells at P3 harvested from a 103 kPa gel and seeded onto a 103 kPa gel) (Fig 8). In the TCP→TCP condition (Fig 8A and S1 Video), within the first 20 min cells adhered to the TCP surface. Within 120 min seeding, mostly all cells had elongated and spread. In the TCP→103 condition, some cells started spreading only at 240 min (Fig 8B and S2 Video), but that changed to 20 min for the cells pre-conditioned to the 103 kPa gels (103→103; Fig 8C and S3 Video). For the TCP→1 condition, we noted that at 300 min post-seeding cells showed minimal signs of spreading (Fig 8D and S4 Video), while conditioned cells began to display spreading at 120 min (1→1; Fig 8E and S5 Video).

**Fig 9:**
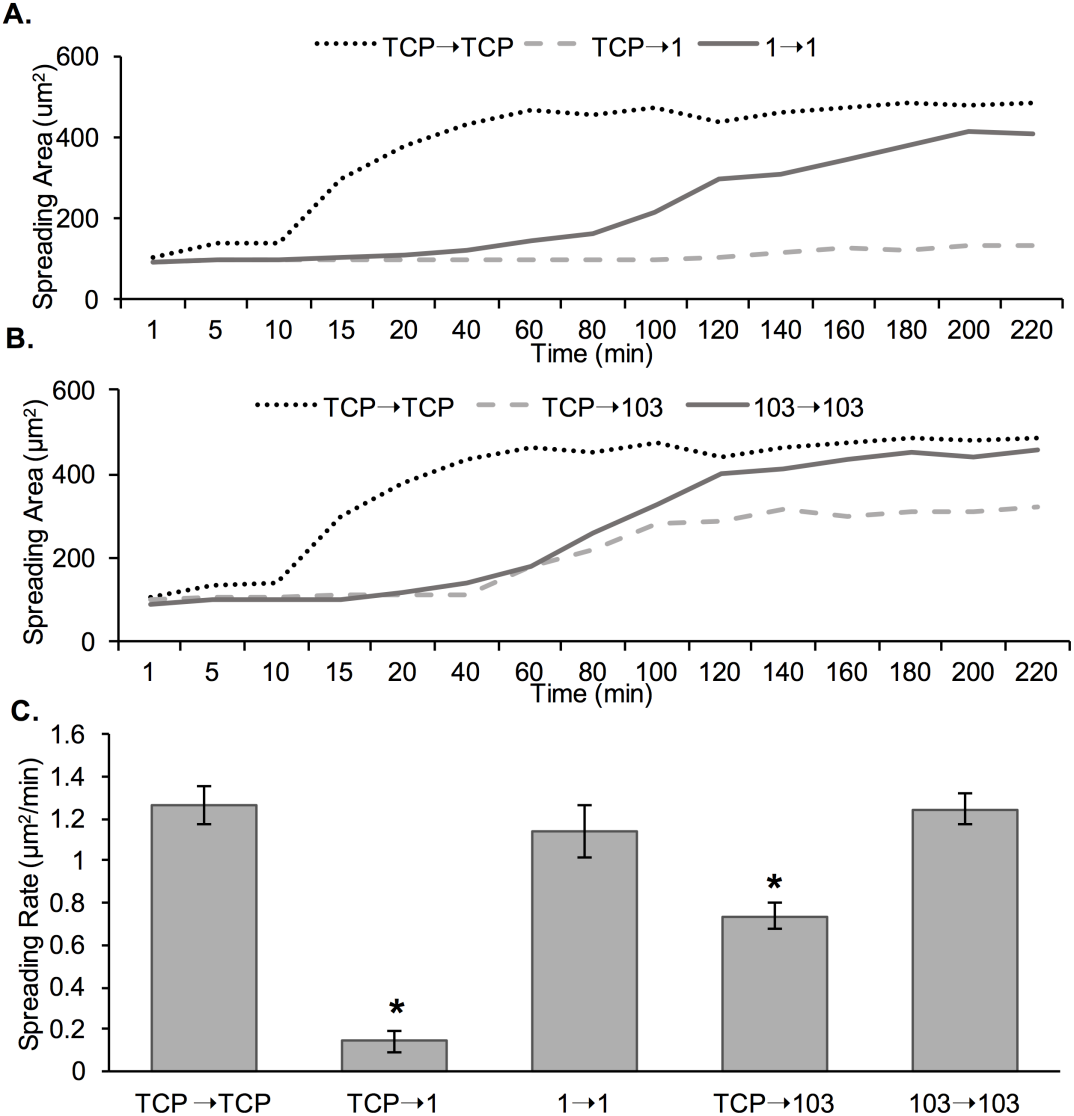
MDA-MB-231 cell spreading area as a function of time and cell pre-adaptation to the substrate. (A) Spreading area for cells seeded onto 1 kPa gel, where cells were either harvested from TCP (TCP→1) or pre-adapted on a 1 kPa gel for 3 passages (1→1). Cells harvested from TCP and seeded onto TCP were used as control (TCP→TCP). (B) Spreading area for cells seeded onto 103 kPa gel, where cells were either harvested from TCP (TCP→103) or pre-adapted on a 103 kPa gel for 3 passages (103→103). Cells harvested from TCP and seeded onto TCP were used as control (TCP→TCP). **C)** Cell spreading rate calculated as per Eq 5. *represent significant difference from all other conditions (p<0.05, n = 3).

To quantify cell spreading rate, spreading area was measured at the various time points and plotted as a function of time for all conditions (Fig 9). Overall, we noted a much shorter lag period prior to cell spreading, steeper slope and earlier saturation for curves (spreading area *versus* time) corresponding to cell on TCP compared to cells on all other hydrogel conditions (Fig 9A and 9B). Comparing between the non-conditioned (TCP→1 and TCP→103) and conditioned (1→1 and 103→103) cells on both 1 and 103 kPa gels, cells started spreading earlier and reach an overall higher spreading area at saturation for the conditioned *versus* the non-conditioned cells; the difference was more pronounced for cells on the soft 1 kPa gels. Also, cells on the 1 kPa control condition (TCP→1) did not show increase in spreading area during the time frame of the experiment. We further quantified the cell spreading rate as per Eq 5 (Fig 9C). We observed a significant 8-fold increase in cell spreading rate between the TCP→1 and 1→1 conditions, indicating that cells were spreading faster on the soft substrate upon conditioning. There was a less pronounced 2-fold increase in cell spreading rate between the TCP→103 and 103→103 conditions, again indicating cell adaptation to the substrate.

### MCF-7 cells exhibit changes in cell morphology and spreading rate upon prolonged passaging on PA gels

**Fig 10:**
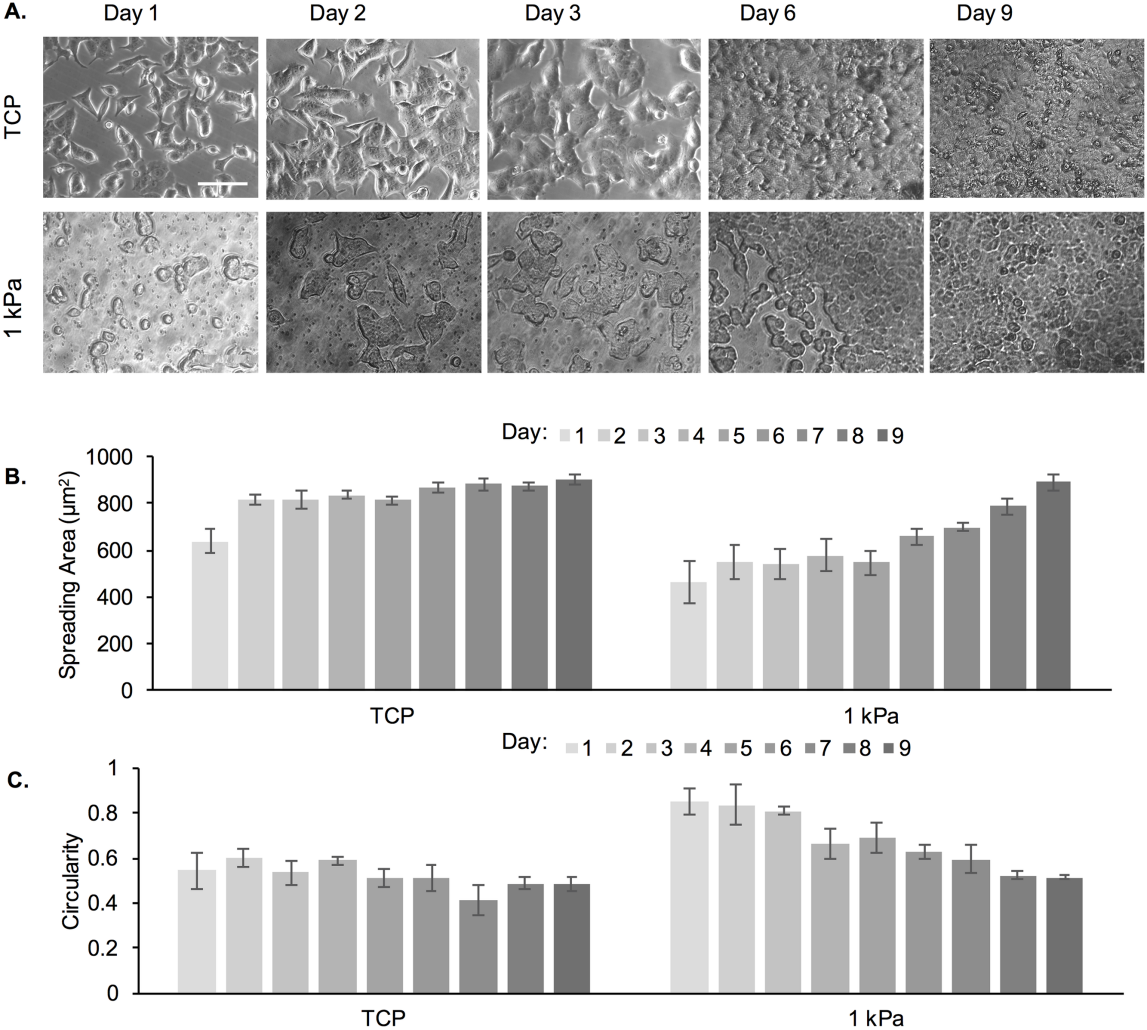
Cell spreading area and circularity of MCF-7A cells as a function of adaptation to substrate stiffness. Cells were seeded onto the PA substrates for 9 consecutive days without re-passaging. (A) Representative phase contrast images of cells on TCP and 1 kPa PA gels. Scale bar is 100 μm. (B) Cell spreading area and (C) circularity of cells on TCP and 1 kPa PA gels as a function of culture time.

We tested a second breast cancer cell line, namely MCF-7 cells, in order to determine if cell changes upon continuous passaging were specific to the MDA-MB-231 cell line. We chose these two cell lines because they have different characteristics and are used extensively in breast cancer research. MDA-MB-231 is the most commonly used model of aggressive metastasis and MCF-7, while non-invasive, is the oldest and most commonly used breast cancer cell line in the world [28]. A continuous 9-day study was conducted; only 1 kPa PA gels and TCP substrates were used (Fig 10). Cells exhibited mostly spread morphologies on day 1 after seeding onto TCP, but not on 1 kPa gels (Fig 10A). MCF-7 cells cultured on TCP had spindle morphology, whereas those on 1 kPa gels displayed a rounded and aggregated morphology with intimate cell–cell contacts. By further quantifying cell spreading area and circularity, we observed no change on TCP as a function of time in culture but an increase in spreading area and decrease in circularity with culturing on the 1 kPa gels (Fig 10B and 10C). Note also that cells reached confluence prior to d 9 (the end of the experiment), which could have contributed to the lack of distinguishable difference between TCP and the 1 kPa gels at d 9 [14].

**Fig 11:**
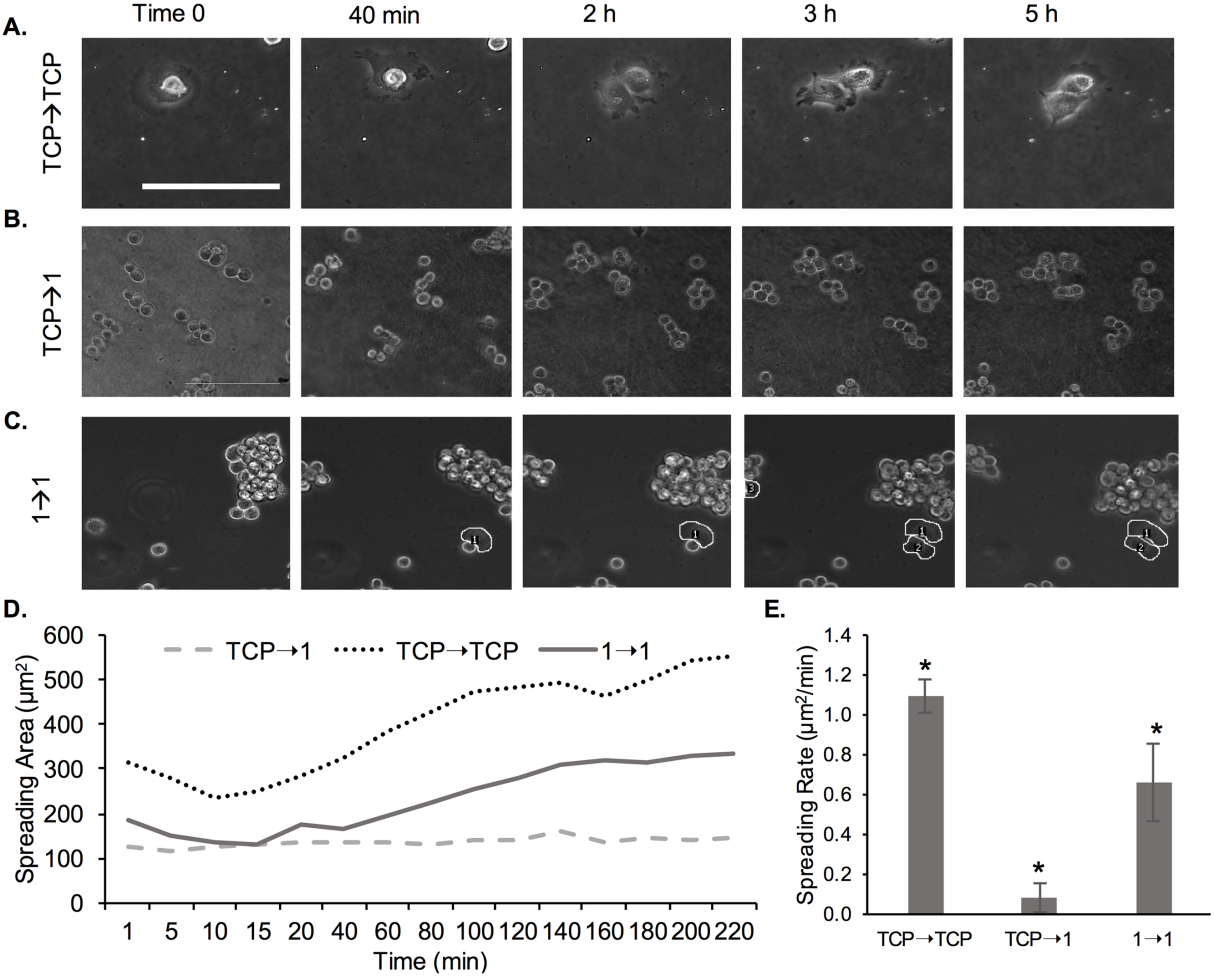
Live imaging of MCF-7 cells on TCP and 1 kPa PA gels immediately post-seeding on the substrate. (A) Cells were harvested from TCP and seeded onto TCP. (B) Cells were harvested from TCP and seeded onto a 1 kPa gel. (C) Pre-adapted cells at P3 were harvested from a 1 kPa gel and seeded onto a 1 kPa gel. Scale bar is 100 μm (**A-C**). (D) Spreading area for cells seeded onto 1 kPa gel, where cells were either harvested from TCP (TCP→1) or pre-adapted on a 1 kPa gel for 3 passages (1→1). Cells harvested from TCP and seeded onto TCP were used as control (TCP→TCP). (E) Cell spreading rate calculated as per Eq 5. *represent significant difference between all conditions (p<0.05, n = 3).

We further conducted live imaging of MCF-7 cells to determine spreading rate (Fig 11). Specifically, we tested spreading of cells harvested from TCP and passaged onto TCP (TCP→TCP), cells harvested on TCP and passaged onto 1 kPa gels (TCP→1) and cells harvested from 1 kPa gels at P3 and passaged onto 1 kPa gels (1→1). Fig 11A (S6 Video) shows increased spreading of cells on TCP over time in which cells took 5 h to exhibit elongated morphologies, but cell attachment was observed within 20 min. Conversely, cells were still circular even at 5 h post-seeding for the TCP→1 condition (Fig 11B and S7 Video). For the 1→1 condition we noted that ~20% of the cells were exhibiting some degree of spreading at 5 h (Fig 11C and S8 Video).

To quantify cell spreading rate, spreading area was again measured for the various time points and plotted as a function of time for all conditions (Fig 11D). Overall, we noted a much shorter lag period prior to cell spreading, steeper slope and earlier saturation for curves (spreading area *versus* time) corresponding to cell on TCP compared to cells on the 1 kPa gels. Comparing between the non-conditioned and conditioned cells on 1 kPa gels (TCP→1 *versus* 1→1), cells started spreading earlier and reached a higher spreading area at saturation for the conditioned *versus* the non-conditioned cells; non-conditioned cells did not show a measurable increase in spreading area during the time frame of the experiment. We further quantified the cell spreading rate as per Eq 5 (Fig 11E). We observed a significant ~7-fold increase in cell spreading rate between the 1 kPa conditioned and unconditioned cells.

## Discussion

Much research is conducted on the effect of substrate stiffness on cell behaviors, yet, much of the work has been conducted with cell lines, which have been pre-adapted to supraphysiologic stiffness; the Young’s modulus of a standard tissue culture dish is ~3 GPa [29]. The question then becomes: should cells be re-conditioned to compliant substrates prior to detailed studies on stiffness-related cell behaviors? In this work, we hypothesized that: *i)* cell behaviors would change as a function of a prolonged pre-adaptation to a compliant substrate, and *ii)* upon adaptation the differences in certain cell behaviors on soft *versus* stiff substrates will be minimized. We specifically focused on cell adhesion, proliferation, morphology, and spreading rate. To test our hypothesis, we used PA hydrogels of 1 kPa (soft) and 103 kPa (stiff) in Young’s modulus and two breast cancer cell lines, namely MDA-MB-231 and MCF-7. Both cell lines are routinely used in basic cancer research [28] and are part of the NCI-60 cell line panel on which all new anti-cancer drugs are tested [30]. Because their tissue of origin is the mammary gland, which has a stiffness of ~0.1–1 kPa, our soft gel was chosen to emulate the native tissue stiffness and the stiff gel was closer in mechanical properties to the cancerous breast stroma, which could be multiple-times stiffer than normal breast stroma [31]. TCP was used as a control in all experiments.

While studies on cell adaptation to the rigidity of the substrate have been conducted previously, such studies have not taken into account prolonged adaptation time. For example, it has been shown that fibroblast cells adapt to substrate stiffness by increasing their own modulus, re-organizing their actin cytoskeleton and exerting higher traction forces after only hours of incubation [21]. In fact, fibroblasts alter their modulus to match that of the substrate up to a threshold of 20 kPa modulus, thus, spanning the range of soft tissue stiffness [20]. However, a recent study of normal *versus* cancer thyroid cells on PA gels of varying stiffness (3–40 kPa) demonstrated that only normal cells changed their elastic modulus and dynamic viscosity proportionally to substrate stiffness, but cancer cells showed no such dependence [32]; hence, such adaptations appear to be cell-specific. In general, the current paradigm is that traction forces exerted by cells arise at localized focal adhesions, whose size correlates to the force exerted by cells on the underlying substrate [33]. Focal adhesion proteins include focal adhesion kinase (FAK), Src family kinases, paxillin, α-actinin, vinculin and talin, which mostly bind to the actin cytoskeleton [34]. Such mechanosensing leads to a myriad of phenotypical cell changes, including in adhesion, proliferation, morphology and spreading rate [34].

Here, we tested MDA-MB-231 cells on PA hydrogels in a range of stiffness from 0.5 to 103 kPa in Young’s modulus and noted stiffness-dependent cell spreading and elongation (Fig 1). Specifically, corroborating earlier studies [11, 15, 18], we noted significant differences between cells on the 1 kPa *versus* the 103 kPa gel in terms of cell spreading and cell shape; hence, we determined that these two gels will allow us to follow adaptations in cell morphology as a function of time. Note that we tested MDA-MB-231 cells on both 0.5 kPa and 1 kPa PA gels, but found no significant difference in cell morphology, thus, we chose to work with the easier-to-handle 1 kPa gel. We chose to focus on cell proliferation, adhesion, spreading, spreading rate and shape, since those properties have been well-documented to be dependent on substrate stiffness [14]. Lastly, we conditioned the cells to the substrate for 9 days, where cells were either passaged every 3^rd^ day for a total of 3 passages or cultured continuously for 9 days to avoid the stress of re-passaging. We chose the 9 day period since we noticed significant changes in cell behaviors at passage 3 or at approximately 6–7 days of continuous culturing on the hydrogel substrates. We chose to include cell re-passaging in addition to continuous passaging for two main reasons. First, cells secrete ECM proteins, thus actively remodeling the underlying substrate over time. Second, cell morphology is regulated by cell-cell interactions in dense cultures in addition to substrate stiffness [35]; hence, we focused on sub-confluent cell layers to decouple the effect of substrate stiffness from cell density.

First, we quantified cell attachment and proliferation as a function of prolonged conditioning to the hydrogel substrates (Fig 2). When cells bind to an extracellular substrate, they recruit various structural and signaling proteins to form focal adhesions, which require mechanical forces to form and mature [36]. Cells on stiff substrates, such as TCP, are able to generate larger forces leading to the formation of mature focal adhesions faster, whereas the same cells on soft substrates cannot provide enough resistance to counter the cell-generated forces [36], leading to less initial attachment of cells on soft substrates. Corroborating such findings, we also noted significantly decreased cell attachment on the 1 kPa gels as opposed to the 103 kPa gels or TCP control for cells at P1 (Fig 2A). We did not attribute these differences to differences in collagen coating of the two substrates of different stiffness. We used the crosslinker sulfo-SANPAH to covalently crosslink collagen to the hydrogels and increase the overall coating efficiency. It has been previously shown by us and others that our method results in even collagen coating for all PA gels and that collagen density is the same for hydrogels of varying stiffness [8, 18]. Within each hydrogel type and TCP control, we didn’t note significant changes in cell attachment efficiency as a function of conditioning to the substrate.

More pronounced differences as a function of conditioning were seen when assessing cell proliferation (Fig 2B). Significantly more cell proliferation was seen both on 103 kPa and 1 kPa gels at P3 compared to P1. However, cell proliferation was still lower on the 1 kPa gel compared to the 103 kPa gel even after conditioning and cell proliferation was lower on the gels compared to the TCP control. Mechanical signaling through the cytoskeleton, which in turn is dependent on substrate stiffness, has been shown to regulate cell proliferation for a variety of cell types [37–39]. For the invasive MDA-MB-231 breast cancer cells in particular, increased proliferation has been noted for matrices of rigidities corresponding to the native tissue rigidities, where the differential rigidity response was related to Fyn kinase signaling [40]. Increased proliferation at 6 days of culture on stiff substrates for MDA-MB-231 and MCF-7 cells has also been linked to more stable focal adhesions and nuclear factor kappa-B (NF-κB) activation [41]. NF-κB is involved in tumor development including cell proliferation and apoptosis [42] and is regulated by actomyosin contractility [43] as well as by cell shape and the microenvironment stiffness in breast epithelial and tumor cells [44]. In a separate study of MCF-7 and T47D breast cancer cells on collagen I-coated PA gels of varying stiffness, the authors linked increased proliferation on stiff substrates to prolactin signaling [45]. Specifically, it was concluded that substrate stiffness shifts the balance of prolactin signaling (implicated in metastatic progression) from physiological to pathological by increasing prolactin signals through focal adhesions [45]. Finally, for breast cancer cells increased proliferation with increased substrate stiffness has been linked to increased invasiveness *via* various proliferative/invasive pathways, such as Rac1 and PI3K [46, 47].

Importantly, when the conditioned cells were harvested from the hydrogels and seeded on TCP, the cells harvested from the 1 kPa gel showed lower cell proliferation compared to cells harvested from the 103 kPa gels (Fig 3), indicating that cell adaptation was not transient; hence the cells exhibited ‘mechanical memory’. Such mechanical memory has recently been shown for lung myoblasts when seeded on polydimethyl siloxane (PDMS) substrates of 5–100 kPa, resembling the stiffness of normal to fibrotic lung tissue [48]. Specifically, when lung fibroblasts were primed on pathologically stiff substrates by continuous re-passaging for 3 weeks, the cells showed sustained myofibroblast activity (hallmark for fibrosis), even when returned to soft cultures for 2 weeks [48]. In another study, the concept of mechanical memory was tested by mechanically ‘dosing’ human mesenchymal stem cells (hMSCs) to influence their fate [49]. The authors used soft (2 kPa) and stiff (10 kPa) polyethylene glycol (PEG) hydrogels as well as supraphysiologic TCP to show that prolonged mechanical dosing—above a threshold period of 10 days, irreversibly biased the stem cells towards osteogenic differentiation [49]. These examples corroborate our findings that cells are able to not only integrate, but store signals from the ECM and also show that an irreversible cell response to substrate stiffness can occur only after prolonged passaging. Very little is known about the underlying mechanisms for such mechanical memory. For example, for myofibroblasts it appears that α-smooth muscle actin (α-SMA) is required, where diminished expression of α-SMA converts myofibroblasts back to MSC-like cells [50]. Another proposed mechanism for MSCs’ mechanical memory is stable chromatin condensation upon dynamic tensile loading, resulting in stiffened nucleus, which persists upon loading cessation [51].

Cell morphology is also highly dependent on the underlying substrate stiffness and previous studies have shown that certain cell lines, including MDA-MB-231, exhibit a more elongated morphology on stiff substrates than on soft ones, which has been related to their ability to generate more traction forces [8, 14, 19]. However, previous studies have mostly shown cell morphology on gels at 1 min to 48 h upon seeding, which might not give cells adequate time to adapt [22, 52, 53]. In this study, we demonstrated that MDA-MB-231 cells were able to adapt to soft gels upon conditioning and displayed higher spreading and more elongated morphologies at P3 as compared to P1 (Figs 4 and 5). At P3 there was no significant difference in cell spreading area or cell shape between the 1 and 103 kPa gels, while the differences at P1 were significant. The differences were more pronounced on the soft 1 kPa than the stiff 103 kPa gels. Similar results were observed when MDA-MB-231 and MCF-7 cells were cultured continuously on a 1 kPa gel (TCP used as control) for 9 days (Figs 7 and 10). By day 6, cells on the soft gels started elongating and spreading, resembling the morphology of cells cultured on TCP. Our results indicate that cells were able to adapt to the soft substrates upon prolonged passaging. Interestingly, cells also seemed to “remember” the stiffness to which they have been pre-conditioned. When MDA-MB-231 cells were harvested from 1 and 103 kPa gels at P3 and seeded onto TCP, they exhibited lower spreading than cells that have been cultured on TCP only (Fig 6).

Lastly, we showed that MDA-MB-231 and MCF-7 cells had initially a lower spreading rate on soft 1 kPa gels compared to stiff 103 kPa gels of TCP. Rapid growth and spreading of cells on stiff substrates and TCP surfaces has been shown by others previously [14, 54]. For example, Yeung *et al*. [14] demonstrated that cells exhibited a 5-times larger spreading rate on stiff 55 kPa gels compared to soft 0.18 kPa gels. Cell adhesion and spreading is mediated by receptor-ligand interactions and has been directly related to traction forces exerted by cells on the substrate. It has been shown that cells exert significant traction forces before focal adhesion or fiber stress formation, whose magnitude is linearly related to the cell area during spreading [55]. However, while cell area and force increase simultaneously during spreading, recent research has shown a delay in cell force development related to spreading area, reflective of the strain-stiffening property of the cytoskeleton [56]. Interestingly, a recent detailed study showed that NIH/3T3 fibroblast cell spreading correlated with substrate stiffness on PA gels, but not on PDMS gels of corresponding modulus (3–1000 kPa) [57]. The difference was explained by the nature of the gels—PA gels are elastic, while PDMS gels behave like viscoelastic solids [57]. A separate study of A549 lung cancer cells on PA and PDMS gels of corresponding bulk stiffness (0.1–40 kPa) also showed that cell spreading rate correlated positively with PA gel stiffness, but was unaffected by PDMS bulk stiffness [58]. The difference in cell spreading was explained by the different interfacial stiffness of the two materials, where interfacial stiffness was found to play a more prominent role compared to bulk material stiffness [58].

We then again tested the concept of mechanical memory of the breast cancer cells by evaluating the cell spreading rate pre- and post-conditioning of the cells on substrates of varying stiffness. Specifically, MDA-MB-231 and MCF-7 cells that have been conditioned to soft substrates (1 and 103 kPa gels) for 9 days and then seeded onto the same substrate, had a higher spreading rate than cells that were transferred from TCP directly onto the gels (Figs 8, 9, and 11). Importantly, for the short observation times of 5 h, we did not see much signs of spreading for the non-conditioned cells on the soft 1 kPa gels, but we observed spreading at 2 h upon conditioning. Note that cells, especially MCF-7 cells, on the 1 kPa gels seemed to stay in clusters prior to adhesion and spreading, which could be due to strong cell-cell interactions and weak cell-matrix interactions [35].

A prior study has shown that rigidity-dependent cancer cell lines, such as MDA-MB-231, growing on soft substrates for 24 h showed reduced spreading and migration, rounded morphologies, and were metabolically less active, indicative of properties of dormant cancer cells [8]. Cancer cells are known to enter a stage of dormancy when introduced to a foreign microenvironment, which has been shown to occur at sites of distant metastases *in vivo* [8, 59]. Similarly, we suggest that when harvesting cells from TCP and seeding them on soft substrates, the cells would enter a state of dormancy; only prolonged passaging would allow cells to adapt to the new environment. Here, we demonstrate that upon adaptation, certain phenotypical differences between cells on soft and stiff substrates diminish or disappear. Importantly, cells remember the mechanical properties of the substrate they were conditioned to, even when subsequently seeded on a substrate of different stiffness. As discussed above, such mechanical memory has been noted previously in lung myofibroblasts and MSCs and has been linked to permanent bias towards fibrotic behavior and osteogenic differentiation, respectively [29, 48].

We focused our adaptation studies on PA hydrogels as they are extensively used to conduct stiffness-dependent cell research [8] and we used breast cancer cells lines. Also, we used collagen Type I as a default coating (since collagen Type I is the most abundant ECM protein found in the human body) [60] to elicit cell attachment, but other ECM proteins such as fibronectin [14, 35] could be used as per the intended application. However, we believe that cell pre-adaptation would be important for other substrates, coatings, or cell types, but that the degree of and time for adaptation will be dependent on the substrate-cell combination used. While not specific to cell adaptation, others have shown that different cell types respond differently to substrate stiffness [18] or substrate coating (i.e. biochemical signals) [61]. Hence, it is reasonable to expect that similar specificity would be applicable to cell adaptation. Thus, it would be our recommendation to establish an appropriate adaptation period for cells when seeded on a biomaterial substrate, in particular cells that have been maintained on a TCP, prior to assessing substrate-dependent cell behaviors.

Note that some cell types might not require adaptation to the compliance of the substrate. For example, certain cell types such as human embryonic kidney cells HEK239, mouse fibroblast cells L929 [62], PC-3 prostate cancer cells [8], or SW620 colorectal adenocarcinoma cells [18] do not show appreciable proliferative or morphological changes in response to substrate stiffness. Also, further work would be needed to establish what other cell adaptations occur upon prolonged passaging on substrates of varying stiffness. There might be adaptation-induced changes in gene expression, cytoskeletal arrangement, motility or drug responses, where changes in response to stiffness have already been documented [11, 14, 19, 63]. The implications of our work and any further findings on cell adaptation would be far reaching as virtually all cell lines have been adapted to TCP and some primary cells are expanded on TCP prior to usage in basic biology research or usage in biomedical devices. In all cases, timing will be critical. As demonstrated by Yang *et al*. [29], short term passaging of MSCs on substrates of supraphysiologic stiffness, such as TCP may lead to reversible changes in cell behaviors, but they become irreversible after a threshold period of time.

## Conclusions

Here, we examined how MDA-MB-231 and MCF-7 breast cancer cells adapt to their environment upon prolonged culturing on PA gels of varying stiffness. Cells cultured for 9 d on compliant PA gels displayed increase in cell spreading area, cell spreading rate, cell attachment and proliferation and decrease in circularity with prolonged time in culture (Table 2). Interestingly, differences in cell behaviors between soft and stiff hydrogels diminished or disappeared with time in culture. Our results indicate that pre-adapting cells to the stiffness of their substrate might be necessary for interpreting stiffness-dependent cell behaviors.

**Table 2:** Changes in cell behavior as a function of prolonged conditioning to the substrate.

Cell Behavior	*Change Post-Conditioning
Spreading Area	↑↑
Circularity	↓↓
Spreading Rate	↑↑↑↑
Cell Proliferation	↑
Cell Attachment Efficiency	↑

*Note: Each arrow indicates 25% change.

## Supporting information

S1 Video Live imaging of MDA-MB-231 cells on TCP. Cells were harvested from TCP and seeded onto TCP.
(MOV)

S2 Video Live imaging of MDA-MB-231 cells on 103 kPa PA gel. Cells were harvested from TCP and seeded onto 103 kPa gel.
(MOV)

S3 Video Live imaging of MDA-MB-231 cells on 103 kPa PA gel. Pre-adapted cells at P3 were harvested from a 103 kPa gel and seeded onto a 103 kPa gel.
(MOV)

S4 Video Live imaging of MDA-MB-231 cells on 1 kPa PA gel. Cells were harvested from TCP and seeded onto a 1 kPa gel.
(MOV)

S5 Video Live imaging of MDA-MB-231 cells on 1 kPa PA gel. Pre-adapted cells at P3 were harvested from a 1 kPa gel and seeded onto a 1 kPa gel.
(MOV)

S6 Video Live imaging of MCF-7 cells on TCP. Cells were harvested from TCP and seeded onto TCP.
(AVI)

S7 Video Live imaging of MCF-7 cells on 1 kPa PA gel. Cells were harvested from TCP and seeded onto a 1 kPa gel.
(AVI)

S8 Video Live imaging of MCF-7 cells on 1 kPa PA gel. Pre-adapted cells at P3 were harvested from a 1 kPa gel and seeded onto a 1 kPa gel.
(AVI)
